# Optimization of Ligninolytic Enzyme Activity and Production Rate with *Ceriporiopsis subvermispora* for Application in Bioremediation by Varying Submerged Media Composition and Growth Immobilization Support

**DOI:** 10.3390/ijms130911365

**Published:** 2012-09-12

**Authors:** Janja Babič, Blaž Likozar, Aleksander Pavko

**Affiliations:** 1 Chair of Chemical, Biochemical and Environmental Engineering, Faculty of Chemistry and Chemical Technology, University of Ljubljana, Aškerčeva 5, 1000 Ljubljana, Slovenia; E-Mail: janja.babic@fkkt.uni-lj.si; 2 Laboratory of Catalysis and Chemical Reaction Engineering, National Institute of Chemistry, Hajdrihova 19, 1000 Ljubljana, Slovenia; E-Mail: blaz.likozar@ki.si

**Keywords:** laccase, manganese peroxidase, ligninolytic enzymes, enzyme production, medium optimization, submerged cultivation, immobilized culture, bioremediation, response surface methodology, decolorization

## Abstract

Response surface methodology (central composite design of experiments) was employed to simultaneously optimize enzyme production and productivities of two ligninolytic enzymes produced by *Ceriporiopsis subvermispora*. Concentrations of glucose, ammonium tartrate and Polysorbate 80 were varied to establish the optimal composition of liquid media (OLM), where the highest experimentally obtained activities and productivities were 41 U L^−1^ and 16 U L^−1^ day^−1^ for laccase (Lac), and 193 U L^−1^ and 80 U L^−1^ day^−1^ for manganese peroxidase (MnP). Considering culture growth in OLM on various types of immobilization support, the best results were obtained with 1 cm beech wood cubes (BWCM). Enzyme activities in culture filtrate were 152 U L^−1^ for Lac and 58 U L^−1^ for MnP, since the chemical composition of this immobilization material induced higher Lac activity. Lower enzyme activities were obtained with polyurethane foam. Culture filtrates of OLM and BWCM were applied for dye decolorization. Remazol Brilliant Blue R (RBBR) was decolorized faster and more efficiently than Copper(II)phthalocyanine (CuP) with BWCM (80% and 60%), since Lac played a crucial role. Decolorization of CuP was initially faster than that of RBBR, due to higher MnP activities in OLM. The extent of decolorization after 14 h was 60% for both dyes.

## 1. Introduction

Fungi are the main organisms responsible for wood biodegradation. Some species of basidiomycetes, the white-rot fungi, are able to efficiently degrade lignin [1]. In order to do that, they produce extracellular oxidative enzymes such as laccase (Lac), lignin peroxidise (LiP) and manganese peroxidise (MnP) [2,3]. Their non-specific oxidative mechanism makes them useful for a wide range of biotechnological applications in the pulp and paper industry and in bioremediation technologies for degradation of recalcitrant compounds [4]. The basidiomycete *C. subvermispora* is a well-known fungus in the bio-pulping and paper industry because it is a selective lignin degrader [5,6]. It is involved in the rare process of selective lignin degradation, where fungal enzymes remove lignin and non-cellulosic polysaccharides without extensive degradation of cellulose itself [6,7]. The decomposition of lignin from various resources may consequently be achieved in an optimal manner and the resulting medium can effectively be utilized as a substrate in bioethanol or biogas producing fermentations following less demanding upstream processing.

Optimization of the culture medium for ligninolytic enzyme production and synthetic dye decolourization using response surface methodology in terms of Lac and MnP activity was first studied by Trupkin *et al.* with the wood-decay fungus *Trametes trogii* [8]. The Plackett–Burman experimental design was utilized to evaluate nutritional requirements [9]. In recent years, the pertinent Lac and MnP activities were subsequently optimized using response surface methodology for white-rot wood-decay and other fungi as well, such as *Pleurotus ostreatus* [10,11], *Trametes versicolor* [12], *Panus tigrinus* [13], *Bjerkandera adusta* [14]. Lac and MnP were predominantly produced using submerged liquid fermentation [8–14]

Filamentous fungi have a natural inclination to adhere to surfaces. This can be successfully utilised in various types of bioreactors with immobilized biomass, for example in packed bed bioreactors, rotating disc bioreactors and biofilm reactors [15–17]. For enzyme production, the chemical composition of the supporting material offering a surface for growth is especially important because it affects fungal activities. It was found that various types of wood with different physicochemical composition have diverse effects on enzyme production during cultivation of *Ganoderma Lucidum* [18,19]. It was indicated that beech wood as an immobilization support induced Lac production with *Dichomitus squalens* better than polyurethane foam since natural lignin acts as an inducer [20]. In our previous study with *C. subvermispora* it was shown that various ratios of Lac and MnP activities can be induced by varying the media composition during cultivation on beech wood, which was the best wood support compared to pine and oak [21].

The aim of this study was to optimize the ligninolytic enzyme activity and production rate and to test the ability of the enzymes obtained to decolourize various synthetic dyes. The first part of the work was dedicated to the study of the optimization of the liquid media. Response surface methodology was applied to simultaneously optimize the activity of two lignin-modifying enzymes produced by *Ceriporiopsis subvermispora*, namely Lac and MnP. This may allow for the optimization of a batch growth medium to tailor the activities of both enzymes simultaneously. Consequently, for each enzyme, optimization was performed using the same methodology, for both the enzyme activity and its corresponding production rate (productivity), defined as the increase in enzyme activity with process time. In the second part of the study, natural fungal characteristics were used to enhance enzyme production by growing it on an immobilization support. According to the literature data [20,21], beech-wood was used here as a promising inducing material. After maximizing the production rate alone, the best combination of optimized growth medium composition and immobilization support should be chosen in the case of either continuous or chemostat-mode process operation. This process could be applied in order to potentially facilitate the scaling-up of Lac and MnP production to the continuous level on the one hand, while, on the other hand, to keep the growth medium composition relatively simple and well-defined. Therefore, various enzyme inducers and mediators were used as components of the immobilization support to obtain the optimal enzyme efficiency rather than as a part of the growth medium. The enzymes produced were used in a decolorization study of two different synthetic dyes to test the suitability of the proposed two step strategy.

## 2. Results and Discussion

In this study the response surface methodology was used to simultaneously optimize the production of two lignin-modifying enzymes (Lac and MnP) produced by *C. subvermispora*. For each enzyme, the optimization was performed using the same methodology for both the enzyme activity and its corresponding production rate. In order to optimize the model consisting of two factors that needed to be improved simultaneously (the maximal enzyme activity and its maximal production rate) as a single system response, the Objective Functions (*OF*_Lac_ and *OF*_MnP_) for batch experiments were expressed by:

(1) $${OF}_{Lac}=\frac{\left[E_{Lac}\right]}{{\left(\left[E_{Lac}\right]\right)}_{max}}w+\frac{d\left[E_{Lac}\right]/dt}{{\left(d\left[E_{Lac}\right]/dt\right)}_{max}}(1-w)=y_{1,Lac}w+y_{2,Lac}(1-w)$$

(2) $${OF}_{Mnp}=\frac{\left[E_{Mnp}\right]}{{\left(\left[E_{Mnp}\right]\right)}_{max}}w+\frac{d\left[E_{Mnp}\right]/dt}{{\left(d\left[E_{Mnp}\right]/dt\right)}_{max}}(1-w)=y_{1,Mnp}w+y_{2,Mnp}(1-w)$$

where ([*E*_Lac_])_max_ and ([*E*_MnP_])_max_ are the highest values of the experimentally obtained maximal Lac and MnP activities ([*E*_Lac_] and [*E*_MnP_] in U L^−1^) for all settings presented in Table 1, *w* is the weighting factor (*i.e.*, the fraction of the normalized maximal enzyme activity contribution in the overall *OF*), (d[*E*_Lac_]/d*t*)_max_ and (d[*E*_MnP_]/d*t*)_max_ are the highest values of experimentally obtained maximal Lac and MnP production rate (d[*E*_Lac_]/d*t* and d[*E*_MnP_]/d*t* in U L^−1^ day^−1^) for all settings presented in Table 1. *OF*_MnP_ could not be optimized due to the absence of local maxima within reasonable substrate concentration values (the combined measurement and estimation error was too large). Nonetheless, a new Objective Function (*OF*_Lac/MnP_) for batch experiments was defined as:

(3) $${OF}_{Lac/Mnp}=\frac{\left[E_{Lac}\right]}{{\left(\left[E_{Lac}\right]\right)}_{\text{max}}}w+\frac{\left[E_{Mnp}\right]}{{\left(\left[E_{Mnp}\right]\right)}_{\text{max}}}(1-w)=y_{1,Lac}w+y_{1,Mnp}(1-w)$$

**Figure 1:**
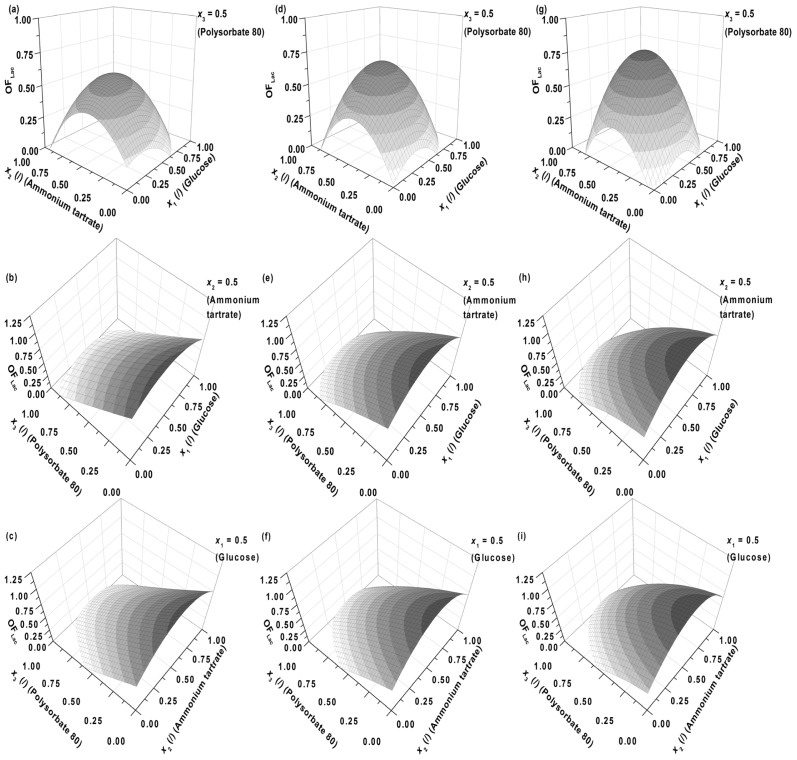
Estimated response surface for *OF*_Lac_ (the contribution of relative laccase (Lac) activity to *OF*_Lac_ (*w*) was set at (**a**–**c**) 0.25, (**d**–**f**) 0.50 and (**g**–**i**) 0.75), on varying the initial glucose (*x*_1_), ammonium tartrate (*x*_2_) and Polysorbate 80 (*x*_3_) concentrations for batch experiments at a temperature of 30 °C.

In order not to introduce too many variables to the design of experiments, as the three crucial independent variables, pertinent to enzyme production, two principal nutrients (carbon and nitrogen source—glucose (1) and ammonium tartrate (2), respectively) and Polysorbate 80 (3), a non-ionic surfactant and emulsifier, were chosen. In Figure 1, the influence of medium components specifically; glucose (*x*_1_), ammonium tartrate (*x*_2_) and Polysorbate 80 (*x*_3_), on the combined activity and productivity of Lac (*OF*_Lac_) is presented. In Figure 1a–c the Lac activity has a lower contribution (0.25) to the overall objective function (Equation 1) than the Lac productivity (0.75), whereas its impact gradually increases through Figure 1d–f (*w* = 0.50) and 1g–i (*w* = 0.75).

The first observation which can be drawn is that the general shape of the response surfaces remains approximately the same, regardless of the fraction of the Lac activity (*w*) and productivity (1 − *w*) in the overall response (*OF*_Lac_), the latter of course being dependent on *w*. Correspondingly, there is an optimum in Lac activity ([*E*_Lac_])/productivity (d[*E*_Lac_]/d*t*) at the specific glucose (*x*_1_) and ammonium tartrate (*x*_2_) concentrations at the fixed Polysorbate 80 level (*x*_3_ = 0.5, *C*_P_ = 0.55 g L^−1^), meaning that at lower concentrations both medium components promote enzyme production, whereas an additional increase in concentrations results in an inhibitory effect. The research of *C. subvermispora* in biotechnological applications revealed the obtained Lac activities around 75 U L^−1^ and MnP activities around 40 U L^−1^ after 5 and 14 days of incubation, respectively, in a MPG (malt extract, peptone, and glucose) medium at 25 °C [22]. Enzyme production by *C. subvermispora* is regulated by nitrogen and glucose concentrations in the medium [23–25]. With a growth limiting amount of nitrogen source (0.2 g L^−1^), the fungus *C. subvermispora* produces low levels of both enzymes compared to a medium with a sufficient nitrogen source concentration (2.0 g L^−1^). The same pattern was found with a carbon source. The fungus produced high levels of both enzymes (about 500 U L^−1^ of MnP and 400 U L^−1^ of Lac, respectively, between two and three weeks of cultivation at 30 °C) at high carbon concentrations (10 g L^−1^ glucose) and low levels at 1.0 g L^−1^ of glucose in a medium with sufficient nitrogen concentration (2.0 g L^−1^) [23]. The enzyme activities obtained by Ruttimann-Johnson *et al.* were higher in comparison to the ones, presented in this study, nonetheless; the enzyme production rates in this study are comparable if not even higher [23]. For a technological enzyme production process the productivity is of a primordial interest, most frequently it is at least comparably important to the enzyme activity itself. This observation is somewhat different from the one indicated in Figure 1. An explanation might be that the above mentioned authors did not examine the effect of carbon source (glucose) concentration over the entire range, but were limited to either its low or high values. Considering Figure 1a,d,g, one could reach the same conclusion, if only two points were compared on the response surface at a fixed nitrogen source concentration (ammonium tartrate) level (*x*_2_). On the other hand, the experimental conditions were not exactly the same in the present study as in the work reported by Ruttimann-Johnson *et al.* [23], as the latter omitted a surfactant in the growth medium. Thus, the concentration of glucose which would suppress enzyme production was not established; nonetheless, it might well exist within their studied range of concentrations of 1–10 g L^−1^ [23] or be even higher. Considering our results, the locus of optimal Lac activity/productivity (*OF*_Lac_) in terms of glucose and ammonium tartrate concentration varies with the contribution of Lac activity to the response, but not significantly (Figure 1a,d,g), implying that medium levels of glucose (2–6 g L^−1^) and tartrate (0.8–1.2 g L^−1^) are favourable for both the activity and productivity of Lac. The latter is pertinent to industrial processes where relatively high yield and rate need to be obtained concurrently. However, extremely high yield and rate cannot be achieved simultaneously, as *OF*_Lac_ in Figure 1a,d,g does not exceed unity, meaning that there is a certain reduction in Lac productivity on account of its higher activity, and vice versa.

**Figure 2:**
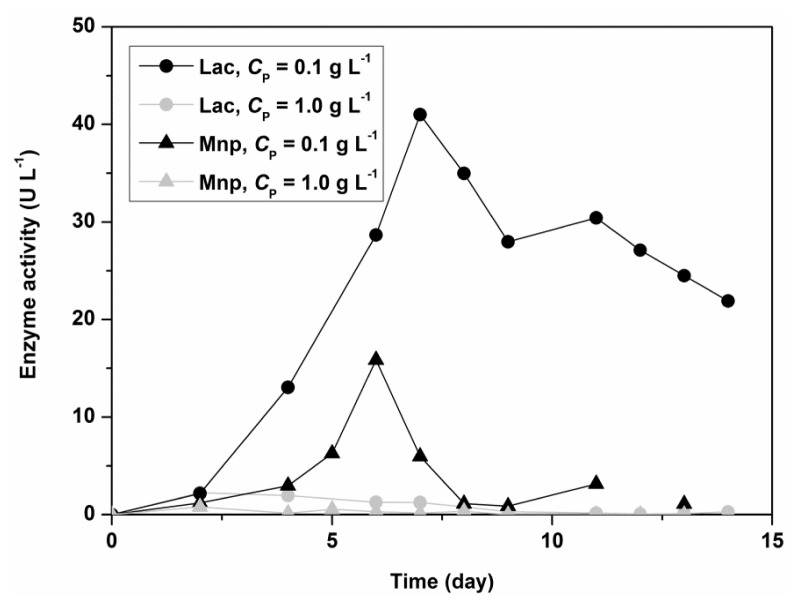
Comparison of laccase (Lac) and manganese peroxidase (MnP) activity obtained from enzyme assays with ABTS (Lac) and DMP (MnP) in batch experiments at a temperature of 30 °C, an initial glucose concentration of 5.50 g L^−1^, an initial ammonium tartrate concentration of 1.05 g L^−1^, and an initial Polysorbate 80 concentration (*C*_P_) of 1.00 g L^−1^ (lower curves) and 0.10 g L^−1^ (upper curves).

In the production of enzymes, especially in large-scale processing, antifoaming agents and surfactants are normally used. Polysorbate 80 (Tween 80) is a non-ionic surfactant that is helpful in releasing fungal enzymes to the external environment. Although Polysorbate 80 is of low toxicity to the cellular membrane, it can alter the structure and morphology of fungi and the bacterial cell wall, leading to an increase of protein secretion [26]. Figure 2 shows the influence of Polysorbate 80 on the activity of Lac and MnP for the fungus *C. subvermispora* in a shaken flask experiment. The initial concentrations of nitrogen and carbon sources were 5.50 g L^−1^ for glucose and 1.05 g L^−1^ for ammonium tartrate. Lac and MnP activities in the filtrate are 10 times higher in the presence of a low concentration of Polysorbate 80 (0.1 g L^−1^). The results thus showed that Polysorbate 80 could be used as a stimulatory agent in submerged fungal cultivation, but in low concentrations. Figure 1b,c,e,f,h,i (model predictions) also show that either at fixed glucose (*x*_1_ = 0.5, *C*_G_ = 5.50 g L^−1^) or ammonium tartrate concentrations (*x*_2_ = 0.5, *C*_DT_ = 1.05 g L^−1^), the concentration of Polysorbate 80 (*x*_3_) should be as low as possible. The dependence on nitrogen or carbon source exhibits a maximum. The latter is again within medium levels of glucose (2–6 g L^−1^) and ammonium tartrate (0.8–1.2 g L^−1^).

The response surface methodology was less successful in describing the activity/productivity of MnP, e.g., the results implied that the optimal MnP activity/productivity would be obtained in the absence of a nitrogen source. Error analysis suggested that this was not a result of the modelling step of the RSM procedure (configure Experimental Section), but of the experimental phase—the production of MnP by *C. subvermispora* was poorly repeatable. However, there was a certain correlation with Lac production, and hence the corrected objective function (*OF*_Lac/MnP_) was defined through Equation 3 for simultaneous optimization of both Lac and MnP activity. In addition to this, even though a poorer predictability of the model for MnP activity and productivity may be observed through lower values of *R*^2^ (the latter indicates explained variability) and *Q*^2^ (the latter indicates predicted variability) coefficients (presented in the continuation), the latter may be considered acceptable. Moreover, the predictability of the new (corrected) Objective Function for the simultaneous optimization of both Lac and MnP activity is more than acceptable on account of excellent predictability for Lac and an average one for MnP.

**Figure 3:**
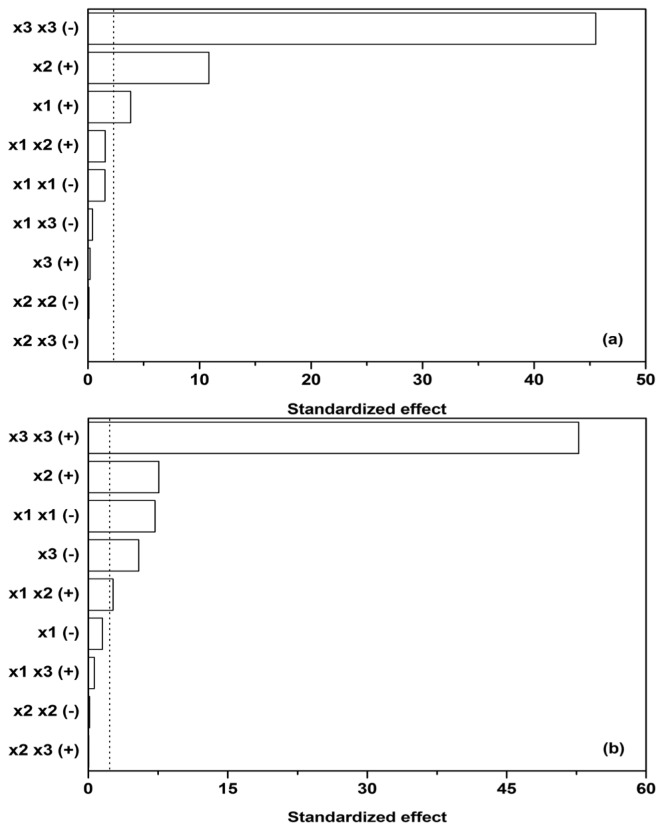
(**a**) Standardized Pareto chart for the maximal Lac activity, [*E*_Lac_]; and (**b**) its maximal production rate, d[*E*_Lac_]/d*t* for batch experiments at a temperature of 30 °C; *x*_1_ is the initial glucose concentration; *x*_2_ is the initial ammonium tartrate concentration and *x*_3_ is the initial Polysorbate 80 concentration. Dotted line represents the 95% confidence interval margin.

Subsequently, sensitivity analysis was performed on the quadratic model utilized and its corresponding parameters (the parameters in Equation 2 and Table 2), estimated by linear regression. Cohen’s *f*^2^ was used to measure the effect size of individual contributions in the model (*x*_1_, the initial glucose, *x*_2,_ ammonium tartrate, and *x*_3_, Polysorbate 80 concentration, *y*_1_, *y*_2_, *y*_3_, and *y*_3_ dependent variables), while the *F*-test was used as a statistical test to determine which effects were within the 95% confidence interval margin (dotted line in Figure 3).

Figure 3a reveals that glucose as a carbon source (*x*_1_) and ammonium tartrate as a nitrogen source (*x*_2_) both exhibit a positive effect on Lac activity (*y*_1_, [*E*_Lac_]), the latter larger. The effect of the Polysorbate 80 quadratic term (*x*_3_ *x*_3_) on Lac activity, on the other hand, is negative and predominant. The quadratic term would imply a stationary point in the dependence of [*E*_Lac_] on *x*_3_, but Figure 1b,c,e,f, 1h, and 1i show no such occurrences. The stationary point thus probably lies outside the presented range, and therefore the joint effect of the linear and quadratic term of *x*_3_ could have been approximated by linearization. Another feature of interest lies in the fact that the influences of carbon source, nitrogen source, and surfactant seem to be independent, as the interdependence terms (*x*_1_ *x*_2_, *x*_1_ *x*_3_, and *x*_2_ *x*_3_) in Figure 3a are negligible, the synergistic effect of glucose and ammonium tartrate (*x*_1_ *x*_2_ (+)) being the largest of them. The dependences on glucose (*x*_1_) and ammonium tartrate (*x*_2_) in Figure 1 nevertheless reveal local maxima.

The maxima in Lac activity/productivity (*OF*_Lac_) as a function of glucose (*x*_1_) and ammonium tartrate (*x*_2_) concentrations in Figure 1 may therefore be predominantly ascribed to the contribution of Lac productivity (*y*_2_, d[*E*_Lac_]/d*t*). Lac productivity is thus noticeably affected by the inhibitory effect of glucose (*x*_1_ *x*_1_ (−)) and the positive combined glucose/ammonium tartrate effect (*x*_1_ *x*_2_ (+)) (Figure 3b). The latter two, along with the linear terms, are responsible for the maxima in the Lac activity/productivity. The effect of ammonium tartrate on Lac productivity is analogous to that which it has on its activity; specifically, it is positive and notable in its influence. Lastly, Polysorbate 80 (*x*_3_) principally affects productivity, as well as activity; the productivity both in the linear (−) and quadratic (+) terms. The overall effect of the surfactant on productivity is the same as that which it asserts on activity, namely negative. Other effects had negligible influence.

The optimization of the quadratic model utilized ensued, where the model parameters were estimated by linear regression. The objective function, constituted from enzyme (Lac and MnP) activity and production rate, was maximized for variable portions of either, *w*. Parameters (the optimal initial medium concentrations *x*_1_, *x*_2_, and *x*_3_) were estimated by nonlinear regression, namely the Levenberg–Marquardt algorithm (10^−5^ tolerance).

**Figure 4:**
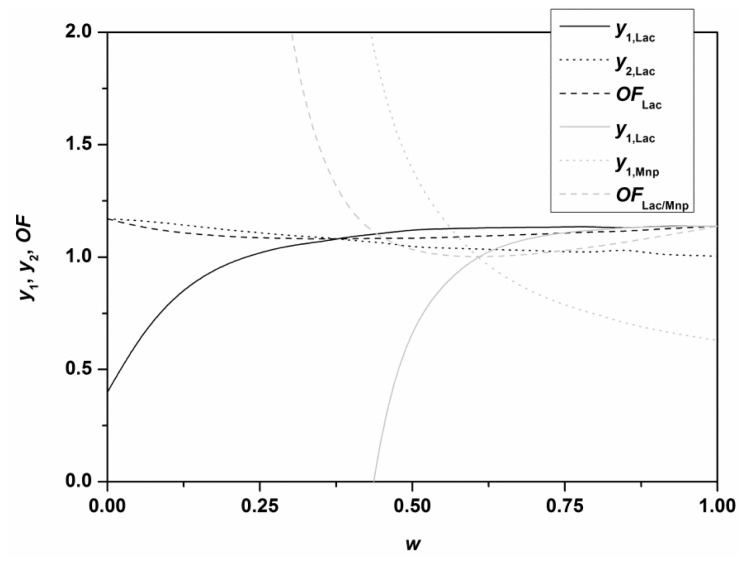
Graph of optimal normalized highest values of experimentally obtained Lac and MnP activities (*y*_1,Lac_ and *y*_1,MnP_), optimal normalized highest values of experimentally obtained Lac production rate (*y*_2,Lac_) and optimal objective function (*OF*_Lac_, and *OF*_Lac/MnP_) plotted against the contribution of the maximal enzyme activity divided by its highest experimental value (*w*).

Figure 4 shows the optimal values of two objective functions, (*OF*_Lac_) Lac activity and productivity, and (*OF*_Lac/MnP_) Lac and MnP activities, which were maximized simultaneously, with variable contributions of Lac activity (*w*) to the objective function. In the first case one may observe that although the overall objective function *OF*_Lac_ assumes its greatest value (1.17) at *w* = 0 due to high Lac productivity, Lac activity is greatly decreased (only 40% of the highest experimental value). As the contribution to Lac activity in *OF*_Lac_, *w*, increases, Lac activity (*y*_1,Lac_) first abruptly and then gradually increases towards 1.14, while concurrently Lac productivity (*y*_2,Lac_) exhibits a rather small decrease, from 1.17 to 1.00. This would imply that, with the exception of very rare cases when the maximal productivity would be attained regardless of the relatively low activity (*w* = 0, *OF*_Lac_ = 1.17), continuous systems should preferably be operated in conditions of the highest activity and only slightly decreased productivity (*w* = 1, *OF*_Lac_ = 1.14), or at least in this vicinity. Of course, another aspect to consider is the concentration of medium components at which distinct levels of activity or productivity may be achieved.

Simultaneously optimizing Lac and MnP activities (*OF*_Lac/MnP_) reveals that it is difficult to obtain high levels of both Lac and MnP, respectively, since for high MnP concentrations (*w*→0), Lac activity decreases basically to zero. On the other hand, upon obtaining high Lac concentrations (114% of the highest experimental value), MnP activity may be maintained at an intermediate level (63% of the highest experimental value).

**Figure 5:**
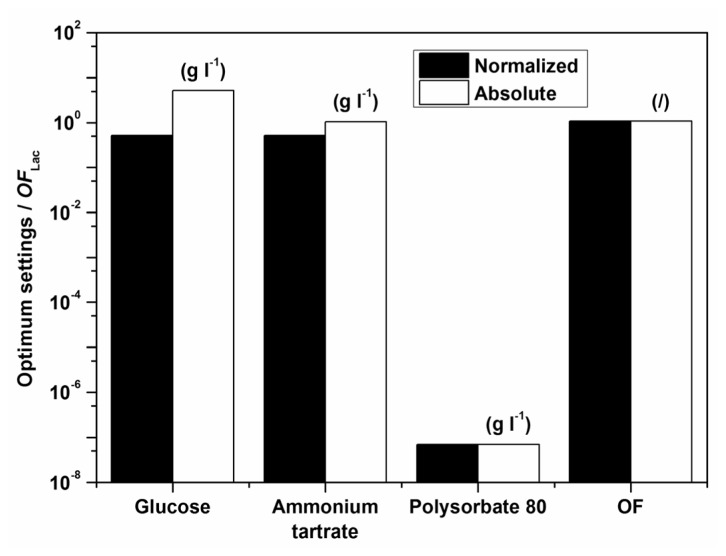
Normalized and absolute optimum settings and *OF*_Lac_ at *w* = 0.5 for batch experiments at a temperature of 30 °C; the maximal enzyme activity and productivity (*OF*_Lac_) are to be obtained after 4 days.

**Figure 6:**
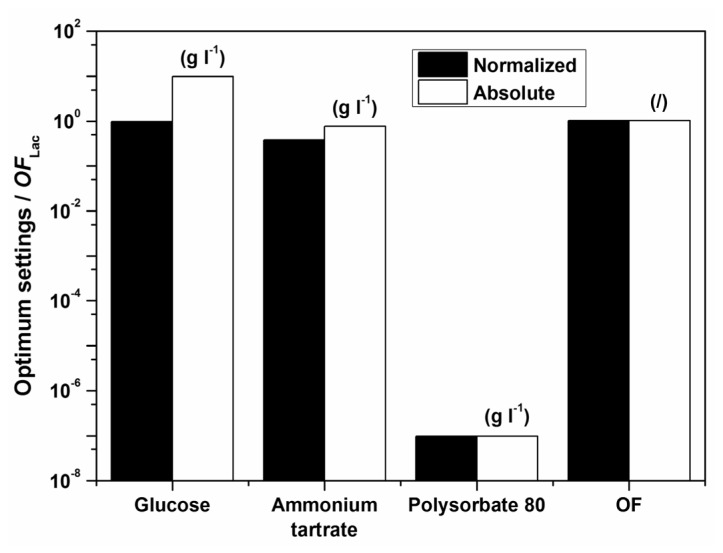
Normalized and absolute optimum settings and *OF*_Lac/MnP_ at *w* = 0.5 for batch experiments at a temperature of 30 °C; the maximal enzyme activities (*OF*_Lac/MnP_) are to be obtained after 4 days.

Examples of initial glucose, ammonium tartrate, and Polysorbate 80 concentrations for the optimal Lac activity and productivity (*OF*_Lac_) and Lac and MnP activities (*OF*_Lac/MnP_) are presented in Tables 3–5 and Figures 5 and 6. In all cases the absence of the surfactant should increase both enzyme activity and productivity. The intermediate concentrations of carbon and nitrogen sources corresponded to the maxima exhibited by the response surfaces in Figure 1. The optimization results had to be verified (Experimental Section) to validate if omitting the surfactant in the medium formulation was indeed favourable for enzyme activity and productivity.

According to the model there should preferably not be any surfactant in the medium. The implication of the fact that the highest Lac activity and productivity are predicted by the model in the absence of Polysorbate 80 is on the one hand favourable, thus avoiding an additional component in the medium and directly decreases cost; furthermore, this could decrease downstream processing and potential contamination of crude filtrates as well. Nonetheless, verification experiments showed a discrepancy between model and experimental data, implying that the range of Polysorbate 80 concentrations examined may have been too broad. Consequently, a series of experiments was undertaken varying only the surfactant concentration in the region below the lowest concentration of 0.3 g L^−1^. The results, which were obtained with optimum settings for *OF*_Lac/MnP_ at *w* = 0.5 (Table 6), clearly indicate an extremely low optimal surfactant concentration, which was determined as 0.05 g L^−1^. Accordingly, the following conclusion may be drawn. The model describes well the dependence of Lac activity and productivity within the examined broad range of concentrations examined; nonetheless it would be prudent to repeat the procedure, illustrated in the Experimental Section, for a narrow window of Polysorbate 80 concentrations in order to achieve better accuracy, especially as far as the decisive surfactant concentration is concerned. Thus, the differences between the measured and the predicted values in Table 6 save for ([*E*_MnP_])_max_ at *C*_P_ = 0 g L^−1^ arise because of the broadly examined range of factors (glucose, ammonium tartrate, and Polysorbate 80 in Table 1)—If parameters were estimated in a narrower range, the optimization would be more accurate; nonetheless, the overall accuracy in the broader range of concentrations would be poorer. Even so, the discrepancy between predicted and experimental laccase production does not confirm the low reliability for this enzyme production, even more the reliability of the predicted laccase production is very high for the studied broad range of nutrient concentrations; only for a more accurate optimization were the additional measurements conducted. In general, the good predictability of the model for Lac activity and productivity may be observed through relatively high values of *R*^2^ (the latter indicates explained variability) and *Q*^2^ (the latter indicates predicted variability) coefficients, which were 0.875 and 0.872 (*R*^2^), and 0.608 and 0.623 (*Q*^2^) for Lac activity and productivity, correspondingly. A poorer predictability of the model for MnP activity and productivity may be observed through lower values of *R*^2^ and *Q*^2^ parameters, which were 0.686 and 0.795 (*R*^2^), and 0.339 and 0.468 (*Q*^2^) for MnP activity and productivity, correspondingly. The objective function (used in the model), using which we aimed to optimize the production, is indeed extremely useful, but in point of fact implies good predictability of the model (e.g., high *Q*^2^). The reported coefficients demonstrate this for Lac activity and productivity, but not for the case of MnP.

**Figure 7:**
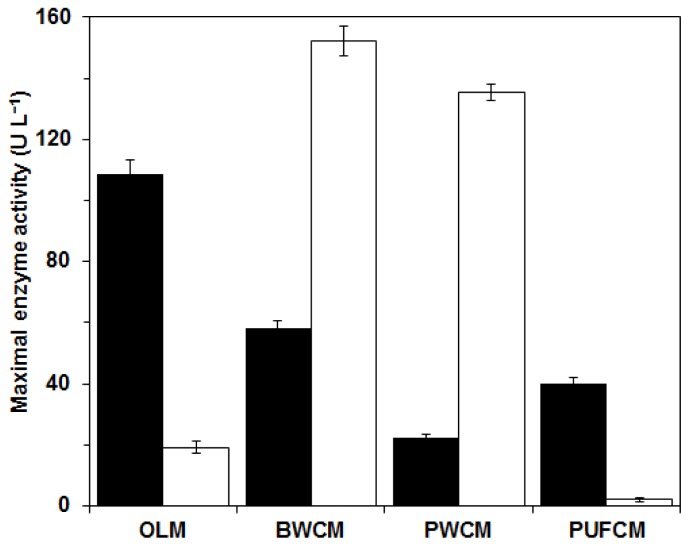
Maximal activities of *C. subvermispora* MnP (■) and Lac (□) in experiments at a temperature of 30 °C with the optimized liquid medium (OLM) and in the medium with beech wood cubes (BWCM), pine wood cubes (PWCM) and inert polyurethane cubes (PUFCM) as immobilization support; the maximal enzyme activities were obtained after eight days.

The second phase of the study continued with *C. subvermispora* cultivation experiments on various immobilization supports using the optimized liquid medium (OLM). Beech wood cubes (BWCM) and pine wood cubes (PWCM) containing natural lignin as inducer and inert polyurethane foam cubes (PUFCM) were used to immobilize the culture growth and to control the immobilization effect in comparison to the combined effect of the other supports that act as carriers as well as inducers. The composition of the liquid media was prepared according to the optimum settings for *OF*_Lac/MnP_ at *w* = 0.5 obtained from the response surface methodology experiment. In addition, the OLM was used as a reference medium with free mycelium. The results are presented in Figure 7. The maximal enzyme activities with free mycelium in the OLM without immobilization support were achieved after eight days, namely 108 U L^−1^ for MnP and 19 U L^−1^ for Lac. It is evident that the use of wood supports affected the enzyme activities. Beech wood and pine wood induced higher Lac activities. Maximal values using BWCM and PWCM, achieved after eight days of cultivation, were 150 U L^−1^ and 135 U L^−1^, respectively. In contrast, MnP activities using wood supports were lower than the results with only liquid medium OLM, namely 60 U L^−1^ for BWCM and 20 U L^−1^ for PWCM. Production of enzymes was the lowest under PUFCM cultivation conditions: MnP activity was only 40 U L^−1^, while Lac activity was close to zero (2 U L^−1^). In the literature it was reported that inert materials such as PUF affect fungal growth as an immobilization surface, but alone cannot increase enzyme synthesis [16,20]. In contrast, natural materials such as straw or wood with structural cell-wall components like cellulose, hemicelluloses and lignin form carbon sources available for fungal growth. In addition, wood contains small amounts of soluble sugars, lipids, peptides and starch as well as minerals and a wide range of extractives and volatiles. All these compounds vary with tree species like pine, beech or oak and therefore affect fungal growth and enzyme production in different ways [27]. Hence, they can be utilized both as an immobilization surface and a natural inducer [20,21]. Present results are also in agreement with our previous investigation, where the synthesis of ‘tailor made’ enzyme composition, that is excess Lac or excess MnP, was proposed by variation of the carbon and nitrogen source in the medium and the use of immobilization supports of different types of wood [21].

**Figure 8:**
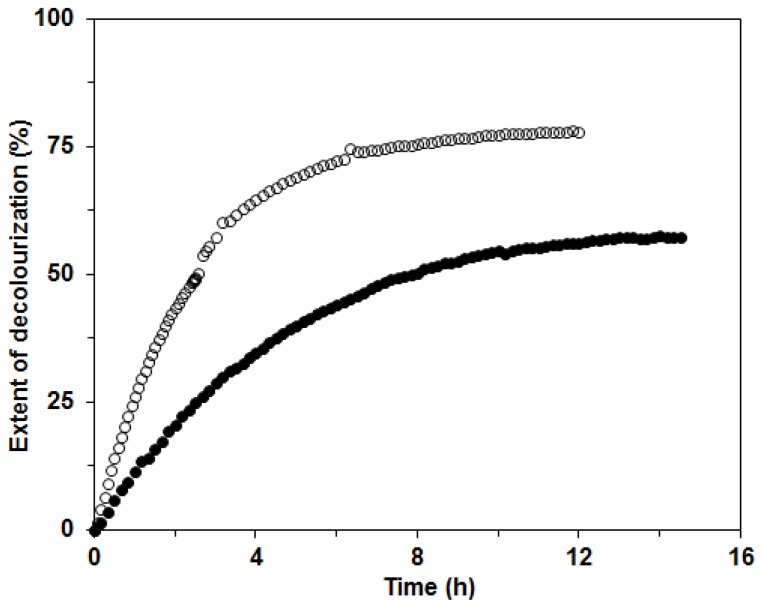
Decolourization of Remazol Brilliant Blue R (RBBR) dye with *C. subvermispora* enzyme mixture from OLM (●) and BWCM (○) at room temperature. Experimental error is in the range of ±4%.

**Figure 9:**
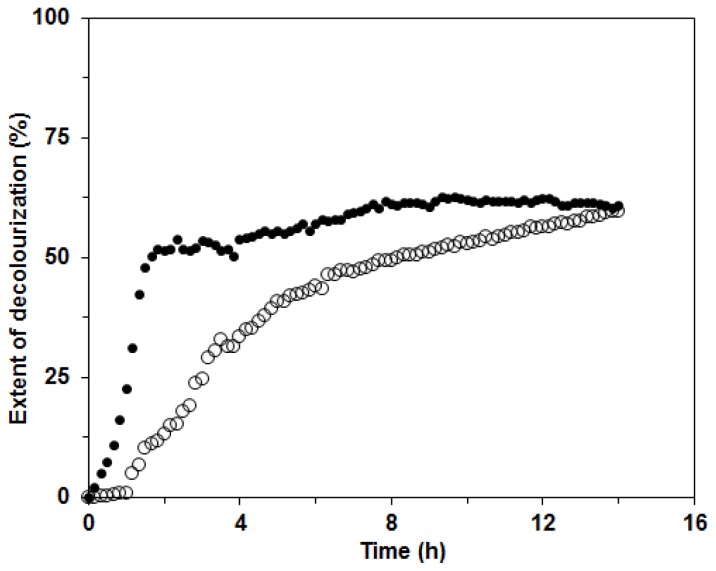
Decolourization of Copper (II) phthalocyanine (CuP) dye with *C. subvermispora* enzyme mixture from OLM (●) and BWCM (○) at room temperature. Experimental error is in the range of ±4%.

Further, the mixture of ligninolytic enzymes produced by the white rot fungus *C. subvermispora* could be used in environmental applications, in the paper industry and also for the decolourization of synthetic dyes [1,4,15,17]. In our investigation, we tested the possible applicability of the ligninolytic enzyme mixture produced in BWCM and OLM for decolourization of the two structurally different dyes Remazol Brilliant Blue (RBBR) and Copper (II) phthalocyanine (CuP). The decolorization of RBBR was faster and more efficient than that of CuP with the enzyme mixture from BWCM (80% and 60% decolorization in approximately 8 and 12 h, respectively). We assumed that Lac played a crucial role in the decolorization of this dye (Figure 8). Our results show that CuP is more difficult to degrade with Lac than RBBR. This effect was already documented during a study of the fungus *Dichomitus squalens* [20]. A similar result, that decolorization of anthraquinonic dyes is easier with laccases produced by white rot fungi, was also reported in the work by Liu *et al*. [28]. The decolorization of CuP was initially faster than that of RBBR, probably due to higher MnP activities in the OLM. When filtrates with lower Lac activities and higher MnP were used for decolorization studies, such as the filtrate from OLM in this study, the compensation and cooperation of these two enzymes in the decolorization process could be implied. These findings were suggested during dye degradation studies with Lac and MnP from *D. squalens* [29]. The extent of decolorization after 14 h was 60% for both enzyme mixtures in BWCM and OLM (Figure 9). The results of our decolorization study with the *C. subvermispora* ligninolytic enzyme system show the potential use of this fungus for environmental protection and bioremediation of pollutants. However, in the future during the enzymatic degradation studies, more attention should be paid to the analysis of the products appearing during enzyme degradation.

## 3. Experimental Section

### 3.1. Microorganism

The fungus strain *Ceriporiopsis subvermispora* CBS 347.63 was obtained from the CBS culture collection (Centraalbureau voor Schimmelcultures, Utrecht, The Netherlands). The strain was maintained on 5% malt extract agar (MEA) at 4 °C as described previously [20].

### 3.2. Batch Experiments

The mycelial suspension was prepared as previously described by Babič and Pavko [21]. The shaken cultures were prepared by inoculating 100 mL of the basal culture medium [30] with 5% (*v*/*v*) of the mycelial suspension. Glucose, ammonium tartrate and Polysorbate 80 concentrations in the basal culture medium were varied as shown in Table 1. The fungus was incubated on a RVI-403 rotary shaker (Tehtnica, Železniki, Slovenia) at 30 °C at a constant agitation of 150 rpm. Aliquots of the liquid culture were collected for determination of extracellular Lac and MnP activities. As well as in the design of experiments in which the central point is repeated, the other experiments were performed in triplicate and consequently with high reliability.

### 3.3. Enzyme Activity

Laccase (Lac) activity was measured by monitoring the oxidation of a 5 mM solution of 2,2′-azino-bis(3-ethylbenzothiozoline-6-sulfonic acid) (ABTS) as the increase in absorbance at 420 nm [31], and manganese peroxidase (MnP) activity by monitoring the oxidation of 20 mM solution of 2,6-dimethoxyphenol (DMP) as the increase in absorbance at 469 nm [32]. One unit of enzyme activity was defined as the amount of the enzyme oxidizing 1 μmol of the corresponding substrate per minute. All spectrophotometric measurements were carried out using a Perkin Elmer spectrophotometer, type Lambda 25 (USA). The substrates for the Lac and MnP activity assays were both purchased from Sigma (USA).

### 3.4. Response Surface Methodology

**Figure 10:**
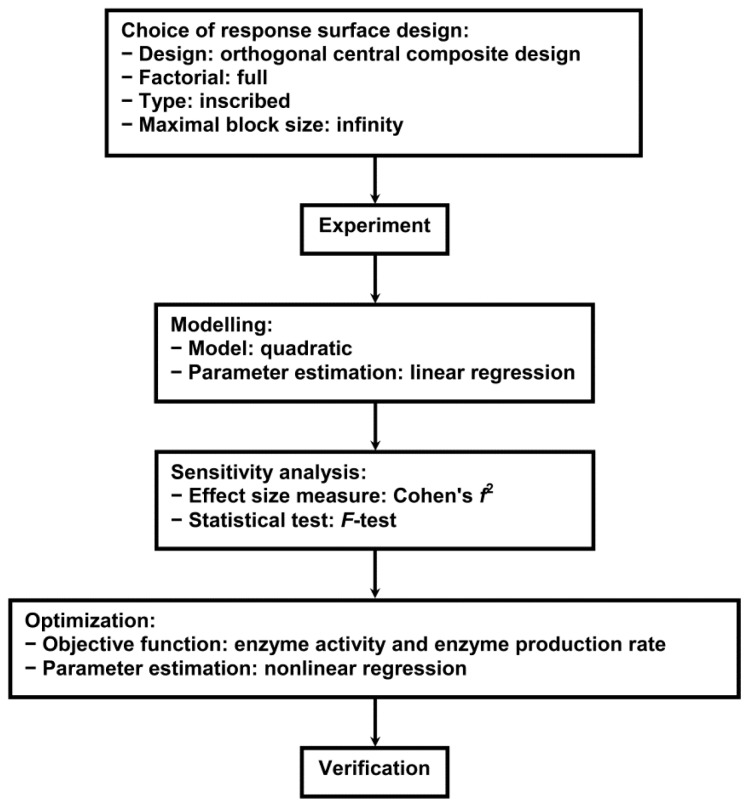
Schematic diagram of the RSM procedure.

To optimize the medium composition with regard to Lac and MnP activity and their productivity, surface response methodology for enzyme activity and productivity was utilized. Central composite design (CCD) (orthogonal) was used applying full factorial, the inscribed type of design, and no limitations of the maximal block size. Although CCD is a really useful approach for the optimization of enzyme production processes it cannot always be successfully applied using fungi; to overcome this possible problem we repeated in triplicate the experiment of the CCD. Modelling, sensitivity analysis and optimization was performed with Matlab R2011a software. The coded values in the central composite design matrix were transformed into real values using minimal and maximal concentration values of 1.0 g L^−1^ and 10.0 g L^−1^ for glucose, 0.1 g L^−1^ and 2.0 g L^−1^ for ammonium tartrate, and 0.1 g L^−1^ and 1.0 g L^−1^ for Polysorbate 80, the real values being presented in Table 1. A schematic diagram of the RSM procedure is laid out in Figure 10, its main steps being response surface design, experiment, modelling, sensitivity analysis, optimization and verification [8–14].

A complete description of the process behavior requires a quadratic model (Equation 4):

(4) $$y_{i}=a_{i0}+\sum_{j=1}^{3}a_{ij}x_{j}+a_{i12}x_{1}x_{2}+a_{i13}x_{1}x_{3}+a_{i23}x_{2}x_{3}+\sum_{j=1}^{3}a_{ijj}{x_{j}}^{2}$$

where *y*_i_ is either the maximal Lac activity (*i* = 1) or its maximal production rate (*i* = 2) and either the maximal MnP activity (*i* = 3) or its maximal production rate (*i* = 4), *a_i_*_0_, *a_ij_*, *a_i_*_12_, *a_i_*_13_, *a_i_*_23_ and *a_ijj_* are respectively, the constant term (*a_i_*_0_), linear (*a_ij_*), mixed (*a_i_*_12_, *a_i_*_13_, and *a_i_*_23_) and quadratic (*a_ijj_*) coefficients for a complete description of the behavior of either the maximal Lac activity (*i* = 1) or its maximal production rate (*i* = 2) and either the maximal MnP activity (*i* = 3) or its maximal production rate (*i* = 4). The coefficients *a_ij_*, *a_i_*_12_, *a_i_*_13_, *a_i_*_23_ and *a_ijj_* correspond to independent variables; specifically, the initial glucose (*j* = 1, *x*_1_), ammonium tartrate (*j* = 2, *x*_2_) and Polysorbate 80 (*j* = 3, *x*_3_) concentrations. Both the independent (*y_i_*) and dependent (*x_j_*) variables were normalized, that is divided by their maximal values, so that they would be within the range of 0–1.

### 3.5. Experiments on Immobilization Supports

Immobilized cultures were grown in optimized liquid medium (OLM) on 1 cm^3^ pine (PWCM), beech (BWCM) and polyurethane (PUFCM) cubes for 14 days. The OLM contained 10 g L^−1^ glucose, 1 g L^−1^ ammonium tartrate and 0.05 g L^−1^ Polysorbate 80 in the basal culture medium. In order to prepare the solid support for fungal colonization, PUF cubes were washed three times with hot distilled water to remove all foreign matter and air dried. The cubes were autoclaved in 150 mL of OLM in 250 mL Erlenmeyer flasks. After autoclaving, 50 mL of the medium was removed and the rest of it was inoculated with 5% (*v*/*v*) of the mycelium suspension. Aliquots of the culture liquid were collected for determination of extracellular Lac and MnP activities.

### 3.6. Dyes and Dye Decolourization

Dye decolourization was calculated by comparing the absorbance, measured at the maximum absorbance wavelength for each compound, during the decolorization treatment. The dyes used in this study were Remazol Brilliant Blue R (RBBR, an anthraquinone dye), λ_max_ = 592 nm, and Copper(II)phthalo-cyanine (CuP; a phthalo-cyanine dye), λ_max_ = 694 nm. Both dyes were purchased from Sigma (USA).

For *in vitro* dye decolorization by the crude culture liquid, culture filtrates obtained from cultivation cultures of *C. subvermispora* in OLM and in BWCM were used. The reaction mixtures consisted of 100 mM Na-tartrate buffer, pH 4.5, 50 mg L^−1^ dye and 100 μL of crude culture liquid in a final volume of 1 mL. Dye decolorization was measured continuously using a Perkin Elmer spectrophotometer, type Lambda 25 (USA) [21].

## 4. Conclusions

In fungal cultures medium optimization should play an important role in the upstream process due to the consequent lowering of process costs. The activity and productivity of ligninolytic enzymes from *C. subvermispora* in submerged cultures and their dependence on medium composition can be well described by response surface methodology (RSM) over a broad range of concentrations. For better accuracy, some repetition of the RSM procedure should be performed.

A two-step strategy, that is, liquid media optimization first and then selection of an appropriate immobilization support to induce the enzyme activities, was successful in increasing Lac activities. It was shown that the methodology of liquid medium optimization can be readily utilized for a wide range of compositions for predicting both ligninolytic enzyme activity and production rate simultaneously and relatively accurately. Therefore, a major advantage of the proposed approach is that it may be usefully applied for systems in which as high activity as possible is desired to avoid downstream separation and purification costs, or for those processes in which maximizing the enzyme production rate is imperative, namely in continuous processes. Balancing these two contributions allows for intermediate regimes in which both the activity and production rate play an important role. Despite the fact that the methodology was applied for a specific medium (glucose, ammonium tartrate, and Polysorbate 80) its applicability may be extended to other components as well.

The natural tendency of filamentous fungi to adhere to surfaces was successfully applied to increase Lac activity in the second step of the study. Naturally an immobilization material with appropriate chemical composition which affects fungal activities and induces enzyme activities should be used for this purpose. This principle, which was used here for enzyme production in batch stationary cultures, is already well recognized for enzyme synthesis in various types of bioreactors with immobilized biomass, for example in packed bed bioreactors, rotating disc bioreactors and biofilm reactors.

It was shown that ligninolytic enzymes, namely manganese peroxidase (MnP) and laccase (Lac), produced by the white rot fungus *C. subvermispora* can be successfully used for decolorization of synthetic dyes. In addition, they also have good potential for use in other bioremediation and environmental protection applications, as well as in lignin breakdown.

## Figures and Tables

**Table 1:** Absolute and normalized factors and settings for response surface methodology (RSM) experiments, and experimentally obtained maximal laccase (Lac) activities and production rates.

Absolute variable (setting) value			Normalized variable (setting) value			Maximal Lac activities and production rates	
		
Glucose (g L^−1^)	Ammonium tartrate (g L^−1^)	Polysorbate 80 (g L^−1^)	*x*_1_ (/)	*x*_2_ (/)	*x*_3_ (/)	[*E*_Lac_] (U L^−1^)	d[*E*_Lac_]/d*t* (U L^−1^ day^−1^)
2.824	0.485	0.282	0.282	0.243	0.282	32.02	10.69
2.824	0.485	0.818	0.282	0.243	0.818	0.88	1.00
2.824	1.615	0.282	0.282	0.807	0.282	20.67	6.64
2.824	1.615	0.818	0.282	0.807	0.818	3.30	1.65
8.176	0.485	0.282	0.818	0.243	0.282	25.99	4.78
8.176	0.485	0.818	0.818	0.243	0.818	2.40	1.20
8.176	1.615	0.282	0.818	0.807	0.282	40.85	8.85
8.176	1.615	0.818	0.818	0.807	0.818	3.24	1.62
5.500	1.050	0.550	0.550	0.525	0.550	21.36	7.64
5.500	1.050	0.550	0.550	0.525	0.550	/	/
5.500	1.050	0.550	0.550	0.525	0.550	29.01	5.34
5.500	1.050	0.550	0.550	0.525	0.550	36.22	7.30
5.500	1.050	0.550	0.550	0.525	0.550	/	/
1.000	1.050	0.550	0.100	0.525	0.550	21.05	8.70
10.000	1.050	0.550	1.000	0.525	0.550	3.15	1.57
5.500	0.100	0.550	0.550	0.050	0.550	1.83	0.65
5.500	2.000	0.550	0.550	1.000	0.550	10.48	4.92
5.500	1.050	0.100	0.550	0.525	0.100	41.02	15.61
5.500	1.050	1.000	0.550	0.525	1.000	2.25	1.12
5.500	1.050	0.550	0.550	0.525	0.550	37.03	7.66
5.500	1.050	0.550	0.550	0.525	0.550	24.58	6.07
5.500	1.050	0.550	0.550	0.525	0.550	37.60	6.45
5.500	1.050	0.550	0.550	0.525	0.550	24.12	4.19
5.500	1.050	0.550	0.550	0.525	0.550	30.38	4.42

**Table 2:** Estimated model parameters for experiments in Table 1.

Parameter	Value (*i* = 1)	Value (*i* = 2)	Value (*i* = 3)	Value (*i* = 4)
*a*_i0_	−0.0999	+1.0141	+0.1965	+1.1103
*a*_i1_	+1.9894	−0.3174	+1.2765	+0.1279
*a*_i2_	+2.2481	+0.8595	−0.3477	−0.7177
*a*_i3_	+0.1828	−1.6619	−0.9645	−2.2866
*a*_i12_	+0.9928	+0.8368	−1.1417	−0.3302
*a*_i13_	−0.5400	+0.4326	−1.7656	−1.3103
*a*_i23_	−0.0099	+0.1115	+1.0478	+1.2429
*a*_i11_	−2.1004	−0.5746	+0.4619	+0.7923
*a*_i22_	−2.5276	−1.1845	+0.2354	+0.0892
*a*_i33_	−0.9523	+0.4468	+0.7538	+1.3955

**Table 3:** Optimized response for *OF*_Lac_ at *w* = 0.1 for batch experiments at a temperature of 30 °C with optimum *OF*_Lac_ value of 1.12.

Factor	Normalized	Absolute
Glucose (g L^−1^)	0.198	1.980 g L^−1^
Ammonium tartrate (g L^−1^)	0.445	0.889 g L^−1^
Polysorbate 80 (g L^−1^)	0.000	0.000 g L^−1^

**Table 4:** Optimized response for *OF*_Lac_ at *w* = 0.9 for batch experiments at a temperature of 30 °C with optimum *OF*_Lac_ value of 1.12.

Factor	Normalized	Absolute
Glucose (g L^−1^)	0.592	5.921 g L^−1^
Ammonium tartrate (g L^−1^)	0.562	1.126 g L^−1^
Polysorbate 80 (g L^−1^)	0.000	0.000 g L^−1^

**Table 5:** Optimized response for *OF*_Lac/MnP_ at *w* = 0.9 for batch experiments at a temperature of 30 °C with optimum *OF*_Lac/MnP_ value of 1.09.

Factor	Normalized	Absolute
Glucose (g L^−1^)	0.637	6.371 g L^−1^
Ammonium tartrate (g L^−1^)	0.552	1.104 g L^−1^
Polysorbate 80 (g L^−1^)	0.000	0.000 g L^−1^

**Table 6:** Laccase and manganese peroxidase activities determined at one of the optimal settings (*OF*_Lac/MnP_, *w* = 0.5) for batch experiments at a temperature of 30 °C. Initial concentrations were glucose 9.79 g L^−1^, ammonium tartrate 0.77 g L^−1^, and Polysorbate 80 0.0–0.3 g L^−1^. Variation among parallel measurements was lower than 5%.

*C*_P_ (g L^−1^)	Measured values		Predicted values	
	
	([*E*_Lac_])_max_ (U L^−1^)	([*E*_MnP_])_max_ (U L^−1^)	([*E*_Lac_])_max_ (U L^−1^)	([*E*_MnP_])_max_ (U L^−1^)
0.00	5.8	89.6	28.5	85.1
0.01	9.2	127.3	28.4	83.7
0.05	9.5	192.8	27.7	78.1
0.20	4.8	146.4	26.7	71.3
0.30	4.5	182.1	20.7	46.4
